# Racing on the Wrong Track

**DOI:** 10.3389/fchem.2017.00042

**Published:** 2017-06-19

**Authors:** Laszlo Otvos

**Affiliations:** ^1^Institute of Medical Microbiology, Semmelweis UniversityBudapest, Hungary; ^2^OLPE, LLCAudubon, PA, United States

**Keywords:** immunostimulation, protein folding, protein synthesis inhibitor, resistance, toxicity

## Abstract

The preclinical *in vitro* and *in vivo* benchmark figures of cationic antimicrobial peptides have to be revisited based on the newly discovered alternative modes of action.

My Pubmed search of the keywords “antimicrobial peptide efficacy mouse” yielded >1,000 citations. Even when I included the further restrictive keywords “systemic infection” that should exclude cutaneous infection models, I still found almost 200 papers. These came from tens of laboratories all focusing on antimicrobial peptide efficacy in mouse models targeting Gram-negative and Gram-positive pathogens, fungi, as well as sepsis, and toxin models. In other words, essentially all aspects of clinical microbiology with the prevailing publication bias already understood, most reporting positive results. Given this robust preclinical interest by the research community one would think that development efforts would be a natural consequence, with clinical trials logically proceeding, in a pace faster than that we saw at the Kentucky Derby, in order to be the first antimicrobial peptide to reach NDA approval in the twenty-first century and establish market dominance. This is not the case, however. As of February, 2015 not a single clinical trial of an antimicrobial peptide against sepsis was registered (Martin et al., 2015), and, other than the decades-old polymyxins, no peptide is on the horizon against bacteremia. In fact, as a research community, we have started to unearth many roadblocks that concern clinical trialists. The more we characterize the mode of action, toxicity, resistance induction, pharmacokinetics and pharmacodynamics, the more exciting questions emerge to which we have little answer, if any at all.

Antimicrobial peptides (or as recently called host defense peptides) have reached NDA approval and late clinical trial stage against nail and skin conditions (Rabanal and Cajal, 2016). One of the reasons is clearly the role of host defense peptides in cutaneous biology and wound healing (Otvos and Ostorhazi, 2015, *vide infra*). Topical treatment therapy can mask systemic toxicity concerns (Bush et al., 2004), although as we very recently documented, cationic host defense peptides can enter the circulation after application to undamaged skin (Ostorhazi et al., 2017).

From the get-go, antimicrobial compounds are evaluated based on their ability to kill various bacterial strains. The desired *in vitro* minimal inhibitory concentration (MIC) threshold values of antimicrobials are strain-dependent and vary based dosage, pharmacokinetics and pharmacodynamics, just mention a few, as well as occasionally they differ in the USA and in Europe (Rodloff et al., 2008). Yet, it is safe to say that regulatory agencies expect MIC values below 2 mg/L (except against very hard to kill bacteria) measured under standard conditions, developed for small molecule drug screening. Almost all native and most designer antimicrobial peptides simply cannot do this. To explain why native peptides can protect insects and other animals from bacterial infection and to convince the industry that we are on to something good, we developed special low salt media in which peptide antibiotics perform better. Indeed, the *in vivo* microenvironment of bacterial growth might be completely different from that in Muller-Hinton broth. Nevertheless, the classic dogma says that cationic antimicrobial peptides kill bacteria by depolarizing of or simply by punching holes in the negatively charged bacterial membrane surface (Yang et al., 2001) and thus low ionic strength can not only influence the efficacy of membrane assembly, but also potentate ionic interactions. In any event, for regulatory approval peptide-friendly media have to be replaced with standard media.

To demonstrate that antimicrobial peptides are as worthy as standard small molecules, we are racing to continuously improve the MIC figures; for cationic peptides this means enhancing a peptide's ability to destroy bacterial cell membranes. To continue the Kentucky Derby analogy, we might be racing on the wrong track. What if the cationic side-chains are just to promote entry into bacterial and host cells? Perhaps antimicrobial peptides have separate domains, one to penetrate cells and another to bind their intracellular target(s); the *in vitro* measure of an MIC would not be reflective of the actual mechanism of action and may falsely be read as activity in the micromolar (cell penetration) concentration rather than the pico- or nanomolar (intracellular targeting) concentration? In this case, we are quantifying something that is completely independent of the particular peptide, and cannot differentiate among peptides that inhibit bacterial nucleic acid or protein synthesis (Hale and Hancock, 2007; Krizsan et al., 2015), protein folding (Kragol et al., 2001) or lipid complexation (McCafferty et al., 1999) just to mention a few non-membrane related but still bacteria-related activities. Then we have to select prudent and universally acceptable measures and benchmarks for alternative modes of action. But if we want to accurately measure the extent of intracellular actions, our assays have little to do with whole bacterial cell survival or proliferation inhibition.

Up to this point, our working hypothesis has been that antimicrobial peptides inactivate something in bacteria. An ever-increasing body of evidence indicates however, that *in vivo*, antimicrobial peptides have stronger effects on host functions rather than bacterial survival with a primary mode host protection grounded in innate immunity activation, at least for peptides close to or under clinical development (Lai and Gallo, 2009; Brandenburg et al., 2012; Hilchie et al., 2013). Perhaps the best example is the remarkable efficacy of the peptide dimer A3-APO and its monomeric metabolite in several mouse infection models when the peptides have very limited bactericidal activity against *Staphylococcus aureus* or *Proteus mirabilis* strains *in vitro*, but significantly improve survival as well as reduce bacterial counts *in vivo* at the infection sites and in the circulation (Ostorhazi et al., 2011). Host responses to cationic peptides include complex immunomodulatory actions (Upton et al., 2012) such as immunostimulation (Wakabayashi et al., 2003), specifically macrophage activation (Welkos et al., 2011), chemotaxis (Radek and Gallo, 2010), or upregulation of anti- or pro-inflammatory cytokine production (Capparelli et al., 2012; Tang et al., 2014). Activation of angiogenesis and other processes instrumental to tissue repair represent the cornerstones of host defense peptide, as adjuvant, use in cutaneous conditions and wound healing with or without bacterial infection (Elsbach, 2003; Bardan et al., 2004; Ostorhazi et al., 2010). Showing efficacy of any given peptide *in vitro*, especially superiority to earlier analogs, requires comparison of a wide range of immune activities, much like comparing apples and oranges; certainly not MICs. *In vivo* we have to resort to improvement of survival and reduction of bacterial loads. Perhaps an accurate measure of pro- or anti-inflammatory cytokine production upon host-defense peptide administration *in vivo* can identify truly outstanding clinical peptide candidates. Luckily a wide range of easy to use ELISA kits are commercially available for this purpose.

The saving grace is that inhibition of bacterial protein folding can attenuate a major bacteria-related health concern, that is, activation of proteinaceous toxin production. A series of bacterial strains express life-threatening polyamide toxins, such as *S. aureus* (α-hemolysin, Berube and Bubeck Wardenburg, 2013), *Clostridium perfringens* (enterotoxin, Freedman et al., 2016), and *Burkholderia pseudomallei* (lethal factor, Cruz-Migoni et al., 2011). Our proline-rich antibacterial peptide dimer A3-APO inhibits *Bacillus cereus* enterotoxin production and *Bacillus anthracis* replication *in vitro*, and statistically significantly delays lethal toxin-induced mortality in a mouse model of anthrax (Otvos et al., 2014a). Luckily, highly accurate bacterial toxin detecting and quantifying kits are commercially available for many of the potential indications and the use of these kits can accelerate drug development. Worth noting, bacteria may still survive upon host defense peptide treatment but they would be unable to produce active proteinaceous toxins.

Along these lines, not only protein-based toxins can be inhibited but any bacterial enzyme that is resistant to conventional antibiotics. Proline-arginine rich antimicrobial peptides can recover the lost activity of legacy antibiotics including β-lactams, chloramphenicol, sulfonamides, or trimethoprim against multidrug resistant strains by inactivating the enzymes that provide resistance against the small molecule antibiotics (Cassone et al., 2008). Antimicrobial peptides may also potentate the *in vivo* effect of legacy antimicrobials through anti-inflammatory effects during classical antimicrobial chemotherapy (Li et al., 2014). Alternatively, synergy *in vivo* may arise between α-helical peptides (Cirioni et al., 2008) or peptides and other antibiotics (Hu et al., 2015) acting on cell wall synthesis through increased drug concentration locally on the target bacterial structures.

One of the perennial arguments for the use of antimicrobial peptides is the lack of resistance induction. This is based on *in vitro* assays in which bacteria are repeatedly incubated with sub-MIC concentrations of antibiotics and then changes in the MIC values are determined after 15–20 passages. This strictly microbiology measure can indeed be useful if the mode of action is only membrane disruption. Host defense peptide resistance is clearly dependent upon membrane activity (Tzeng et al., 2005; Kindrachuk et al., 2007). However, for peptides with alternative modes of action, the sublethal passage assay has little positive predictive value. The major microbiological difference between A3-APO and its monomeric analog, Chex1-Arg20, is the improved membrane-disruptive activity of the dimeric prodrug. Only the monomer induces microbiological resistance, and only against one strain. The intracellular target, however, DnaK, remains preserved after multiple passages with no genetic alterations; the DnaK multihelical lid region, where the peptides bind and the putative transport protein SbmA are unchanged (Cassone et al., 2009). More concerning, *in vitro S. aureus* develops resistance to magainin, with cross-resistance to human-neutrophil-defensin-1, a key component of the innate immune system (Habets and Brockhurst, 2012) projecting potential risks of host defense peptide therapies.

Finally, investigational new drug applications (IND) typically require pharmacokinetic parameters as detailed in published FDA Guidance documents. These parameters, however, may be problematic for antimicrobial peptides. In the classical view, to protect mammals from bacteremia, we need to maintain a sustained (multiple hours) circulating antimicrobial concentration of 1.3 × MIC (Otvos et al., 2005); this may not, as currently measured, be supportive of an antimicrobial peptide IND application. First, positively charged antimicrobial peptides avidly bind negatively charged components of not only bacteria, but also the mammalian body, including serum albumin. To measure the blood level of both bound and free antimicrobials, special chromatography/mass spectroscopy protocols should be used (Schmidt et al., 2016). Second, for alternative modes of action, e.g., to trigger a host immune response, the required circulation levels can be 1,000 times less than that for bactericidal activity and frequently below current detection limits (Otvos et al., 2014b). Third, when peptides bind their targets, the ligand residence time is very long, and the targets remain engaged considerably longer than the time period of typical renal elimination. For peptide drugs, pharmacodynamics (what the drug does to the body) is a more practical measure of biological activity than pharmacokinetics (what the body does to the drug, Otvos and Wade, 2014).

So my fellow host defense peptide riders please take off the blinkers and ride your horses on the correct tracks, sometimes not frequented by other contestants, to win the race. For one, I have not bet for the favorite at the Preakness.

## Author contributions

The author confirms being the sole contributor of this work and approved it for publication.

### Conflict of interest statement

LO is the inventor of an antimicrobial peptide patent owned by Temple University and licensed by Arrevus, Inc. The author is a consultant for Arrevus.
